# Validity and reliability of ChatGPT's responses on dietary supplements in Japan: A quality assessment and content analysis

**DOI:** 10.1016/j.pecinn.2026.100461

**Published:** 2026-02-21

**Authors:** Mingxin Liu, Tsuyoshi Okuhara, Ritsuko Shirabe, Yuriko Nishiie, Xinyi Chang, Hiroko Okada, Takahiro Kiuchi

**Affiliations:** a Department of Health Communication, Graduate School of Medicine, The University of Tokyo, Tokyo, Japan.; b Department of Health Communication, School of Public Health, Graduate School of Medicine, The University of Tokyo, Tokyo, Japan.; c University hospital Medical Information Network (UMIN) Center, The University of Tokyo Hospital, Tokyo, Japan; d Department of Industrial Engineering and Economics, Institute of Science Tokyo, Tokyo, Japan

**Keywords:** ChatGPT, Dietary supplements, Large language models, Misinformation, Health information reliability

## Abstract

**Objective:**

This study evaluated the validity and reliability of large language model (LLM) responses on dietary supplements (DS), a domain marked by scientific controversy and misinformation. The goal was to support informed consumer decisions and guide improvements in LLM performance.

**Methods:**

We collected responses from GPT-4 and GPT-4o on the effects of 30 DS on six diseases. Two medical professionals categorized each response as “Effective,” “Uncertain,” or “Not Effective.” They also created a guideline to assess evidence-based effectiveness and compared it with LLM-generated responses to determine accuracy. Additionally, we conducted qualitative content analysis to identify response patterns and misleading content.

**Results:**

GPT-4 and GPT-4o affirmed DS effectiveness in only 10% of cases, with 40% rated as “Uncertain” and 50% as “Not Effective.” Accuracy was about 57%, considerably lower than that observed in nutrition-related studies (57% in DS vs. 80% ∼ in structured nutrition tasks”). Content analysis showed templated responses, frequent ambiguity, and occasional inclusion of irrelevant or incorrect information.

**Conclusion:**

Our findings suggest that ChatGPT's responses on dietary supplements are generally cautious but often ambiguous, with a moderate risk of misinformation. As generative AI becomes a common source for health advice, these limitations could mislead users. Enhancing LLMs' evidence-based accuracy and ensuring consistent professional guidance are essential.

**Innovation:**

This is the first study to assess the validity and reliability of LLM-generated responses on dietary supplements using both quantitative and qualitative methods. We also developed a novel evidence-based framework to evaluate supplement effectiveness, providing a new tool for future research and supporting safer AI-assisted health communication.

## Highlights

• This is the first study to assess GPT-4 and GPT-4o on dietary supplements.
• Only 10% of responses supported supplements as treatments for specific diseases.
• GPT responses achieved 57% accuracy, lower than in structured nutrition knowledge.
• LLMs achieved highest accuracy for cancer and lowest for constipation topics.
• LLMs often produce ambiguous, lengthy, or expectation-driven responses.

## Graphical abstract

**Figure:**
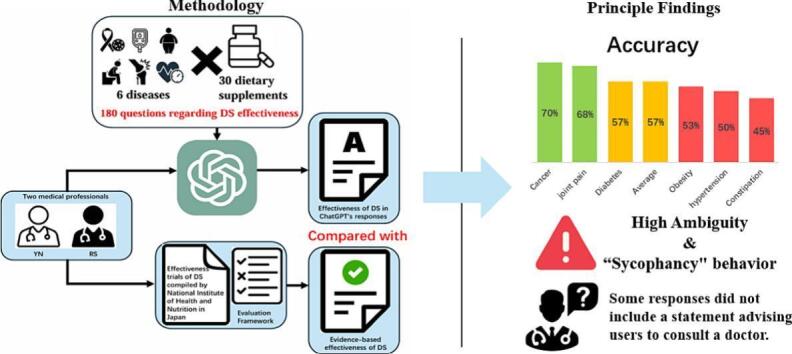

## 1 Introduction

### 1.1 Background

In November 2022, with the release of ChatGPT, large language models (LLMs) quickly gained global attention for providing detailed answers to complex queries [1]. They have since been widely applied across domains such as programming, education, business, and law, achieving notable success [2], [3], [4], [5]. Researchers have also actively explored their potential roles in medicine [6], [7], [8], [9], including recent studies on their feasibility for dietary and nutritional advice.

Most previous studies focused on evaluating ChatGPT as a virtual nutritionist and assessing the accuracy of its dietary responses [10], [11], [12], [13], [14], [15], [16], [17], [18], [19]. Some studies have reported that it achieves over 80% accuracy and demonstrates rational reasoning, outperforming college students and even professionals. [10], [13], [14], [18]. However, in complex scenarios requiring precise knowledge, most studies agree ChatGPT cannot replace human experts [11], [12], [15], [16], [17].

Yet, few studies have examined ChatGPT's performance in the dietary supplement (DS) domain. One editorial assessed ChatGPT 3.5's responses about vitamin A [20], but it only concluded that ChatGPT cannot replace professional consultation, without deeper analysis of its accuracy or flaws. Moreover, vitamin A alone cannot represent ChatGPT's overall performance in DS. We argue that evaluating ChatGPT in this field is necessary for several reasons.

Firstly, DS are regulated less strictly than foods or pharmaceuticals, and their efficacy claims are rarely subject to standardized clinical validation [21], [22]. Second, the scientific evidence surrounding DS is highly heterogeneous, with studies often producing contradictory results depending on formulation, study design, or methodological quality [23]. Third, commercial marketing and influencer-driven promotion frequently shape public perception more strongly than scientific consensus, leading to widespread misinformation. These characteristics make the DS domain a uniquely complex and high-risk area for evaluating the reliability of LLM-generated health information [24]. Accordingly, our study extends beyond conventional nutrition research by examining how advanced LLMs perform in a field where evidence is fragmented, regulatory oversight is limited, and consumer trust is particularly vulnerable.

From the consumer perspective, extensive previous research has shown that most consumers, including students, elders, military personnel, and even health science students, lack sufficient knowledge of DS [25], [26], [27], [28], [29], [30], [31], [32]. This lack of knowledge makes it difficult for consumers to discern the efficacy of these products and make informed choices. DS are subject to less stringent regulations than pharmaceuticals. The Internet is flooded with a large amount of misinformation about DS from individual users or merchants, and the efficacy of DS is also the hardest hit by merchants who exaggerate or falsely advertise them. A study in Bahrain showed that over 80% of the information about DS on social media was incorrect or potentially misleading [33]. Thus, it may be difficult for consumers to obtain accurate information on DS using the Internet. Research has also shown that consumers may recommend specific DS to sellers who are not recommended by medical professionals [34]. Furthermore, consumers tend to visit clinics or hospitals to consult doctors when they are sick rather than searching for treatments and making decisions on their own via the Internet, unlike DS, for which consumers who lack sufficient knowledge are more likely to search for their efficacy and effects on the Internet before making purchase and consumption decisions [35], [36], [37], [38], [39].

As LLMs become more widespread, consumers may use this convenient tool to inquire about the efficacy of DS, thereby influencing their behavior and decisions. Therefore, understanding LLMs' capability of LLMs to provide reliable information on DS is important to public health researchers.

### 1.2 Study aims and objectives

We simulated scenarios in which Japanese individuals without specialized medical knowledge asked generative AI questions on DS, a topic known for its controversial and misleading nature. This study aims to clarify the following issues:

1. What number and proportion of ChatGPT responses indicate whether a DS is effective, uncertain, or not effective in its responses?
2. How do GPT-4 and GPT-4o perform in addressing questions across different disease categories?
3. How accurate are the responses provided by GPT-4 and GPT-4o in the DS domain?
4. What are the common issues in ChatGPT's responses, and how do these issues potentially mislead users?
5. How can the accuracy and reliability of ChatGPT in the field of DS be improved through updates and training?
6. Are there any notable differences in the consistency of responses between GPT-4 and GPT-4o?

By addressing these questions, we evaluated the performance of LLMs in the domain of DS and identified their limitations. This study seeks to provide recommendations for the future development and updating of LLMs, while also offering guidance to the public to help them make informed decisions when seeking information about DS from LLMs.

## 2 Methods

### 2.1 DS keywords and questions

We included the top 30 best-selling DS in Japan market in 2023, as listed in [Chapter 4: Present Situation and Prospects of the Health Food Category Market] of [2023 Edition: Healthy Foods Market Stats and Prospects ∼ Market Survey Edition ∼] published by Yano Research Institute Ltd. (Table 1), were involved in this study [40].

**Table 1:** Keyword of 30 DS.

Aojiru	Agaricus	*Ginkgo biloba* extract
Turmeric	Royal jelly	Ornithine
Oyster Extract	Chlorella	Glucosamine
Chitin & Chitosan	Ubiquinone (UQ)	Chinese softshell turtle
Black Vinegar	Squalene	Collagen
Oriental ginseng	Soy Isoflavone	DHA & EHA
Garlic	Lactic acid bacteria	Hyaluronic acid
Vitamin E	Vitamin C	Placenta
Blueberry & Bilberry	Prune	Propolis
Maca	Euglena	Calcium

The National Institute of Health and Nutrition in Japan has published reports on the effectiveness of hundreds of DS for various body systems (Supplementary Materials 1) [41]. These systems include the circulatory and respiratory systems, digestive and liver systems, diabetes and endocrine systems, reproductive and urinary systems, brain and sensory organs, immune system, cancer and inflammation, bones and muscles, growth and development, and obesity. We corresponded to six common diseases and formulated questions accordingly.

We created six questions for each DS by referring to a report on DS published by the National Institute of Health and Nutrition in Japan (Table 2).

**Table 2:** The six questions asked LLMs.

Question 1	Is [Supplement Name] (e.g., Aojiru) effective against cancer?
Question 2	Is [Supplement Name] (e.g., Aojiru) effective against diabetes?
Question 3	Is [Supplement Name] (e.g., Aojiru) effective against obesity?
Question 4	Is [Supplement Name] (e.g., Aojiru) effective against constipation?
Question 5	Is [Supplement Name] (e.g., Aojiru) effective against joint pain?
Question 6	Is [Supplement Name] (e.g., Aojiru) effective against hypertension?

### 2.2 Tested LLMs and data collection

We entered questions on each DS into the GPT-4 and GPT-4o in Japanese via the ChatGPT web interface and recorded the answers in an Excel spreadsheet. To simulate real-world scenery, we obtained 30 × 6 × 2 = 360 outputs by asking one question per supplement–disease pair to each model (GPT-4 and GPT-4o). Each question was asked once per model in a new dialog, and the first response was recorded without any re-rolling or prompt engineering. We collected responses from GPT-4 between July and September 2023, and responses from GPT-4o in September 2024. Additionally, to ensure the consistency of the study, we checked the cutoff date of the training data of the GPT-4 and GPT-4o to ensure that the study used the same version of the LLM.

### 2.3 Quality assessment

Reports from the National Institute of Health and Nutrition in Japan on each type of DS do not directly state whether a particular supplement is effective against a specific disease. Instead, they compiled all experimental studies that evaluated the effects of this supplement on the disease. These studies encompass a variety of methodologies such as randomized controlled trials (RCTs), meta-analyses, and animal experiments. Studies may present either positive or negative results for the same DS and diseases (Supplementary Materials 1).

**Fig. 1:**
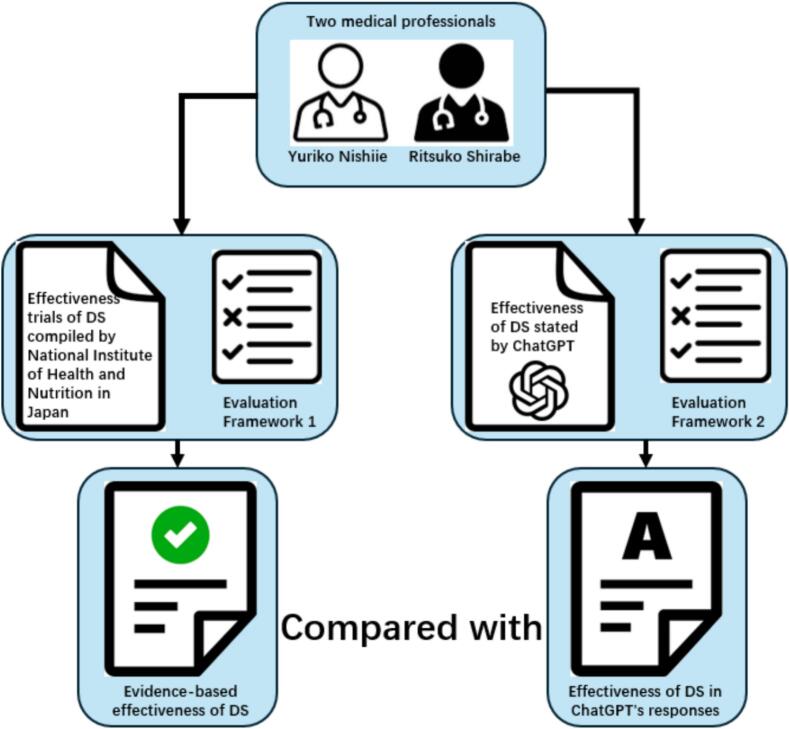
A flow diagram of the method.

To address this variability, two authors with Japanese medical licenses (YN and RS) developed a comprehensive evaluation framework (Supplementary Materials 2). Using this framework and reports from the National Institute of Health and Nutrition in Japan, they were able to assess the effectiveness of 30 DS for six diseases objectively. The outcomes were classified into three categories: “Effective,” “Uncertain” and “Not Effective.” In the case of contradictory results, a resolution was achieved by consensus. We added examples in the Supplementary Materials 2 to illustrate how evidence for one supplement was classified according to these rules. The method flow was shown in Fig. 1.

Subsequently, two physicians (YN and RS) evaluated the effectiveness of DS for specific diseases as presented in the GPT responses using another guideline developed by them (Supplementary Materials 2). The outcomes were also classified into three categories: “Effective,” “Uncertain” and “Not Effective.” This process was conducted in a double-blind manner. The Fleiss' κ was used to evaluate the consistency between the two authors. Discrepancies were resolved through discussion to reach a final consensus. An example in which the two physicians initially disagreed on ChatGPT's assessment of a dietary supplement's effectiveness but reached a consensus through discussion was shown in supplementary materials 2.

To verify the validity of the evaluation framework developed by the two physicians, we conducted a validation pilot study between June and July 2024 using dietary supplements other than those included in the main analysis of this study.

Finally, the evidence-based effectiveness was compared with that of the effectiveness answered by GPT. If the GPT response aligned with evidence-based effectiveness, it was deemed correct; otherwise, it was deemed incorrect. The GPT responses were recorded, and the comparison process was conducted using Microsoft Excel. We conducted **a z-test** to clarify the significance of the differences between groups. In addition to pairwise z-tests, we conducted a chi-square test of independence to evaluate whether the accuracy of GPT responses varied significantly across disease categories (cancer, diabetes, obesity, constipation, joint pain, and hypertension). Furthermore, we fitted a binary logistic regression model, with correctness (correct = 1, incorrect = 0) as the dependent variable and disease category as a categorical predictor. Both analyses were performed in R version 4.3.1, and significance was set at *p* < 0.05.

### 2.4 Content analysis

In addition to counting the accuracy of GPT responses in a quantitative study, this study also used a qualitative method to analyze the content of the GPT responses. We reviewed all 360 GPT responses and documented the notable characteristics and issues observed using Microsoft Excel. We summarized these features of the GPT's responses, as well as common mistakes, and cited relevant excerpts in Japanese, along with their English translations, to illustrate and support our observations. Two authors (ML and XC) independently reviewed all responses, and any discrepancies were resolved through discussion to reach consensus. Percentages reported represent the proportion of responses within each code category relative to the total number of responses. Through a content analysis, we provide detailed recommendations for both AI developers and users.

## 3 Results

### 3.1 Evidence based effectiveness of DS

The results of the evaluation of the evidence-based effectiveness of the 30 DS for the six diseases are presented in supplementary materials 3. The “A,” “B” and “C” indicate “Effective,” “Uncertain,” and “Not effective,” respectively. Most DS have either no or uncertain effects on the disease.

### 3.2 Proposed effectiveness of DS in GPT responses

The Fleiss' κ values for the pilot study and the main study were 0.75 and 0.72, respectively, indicating a high level of inter-rater consistency. Table 3 shows the number of “Effective,” “Uncertain,” and “Not effective” responses from GPT-4 and GPT-4o regarding 30 DS for 6 diseases. GPT-4o and GPT-4 gave responses indicating the effectiveness of supplements for diseases 18 (10.0%) and 20 (11.1%) times, “Uncertain” responses 68 (37.8%) and 76 (42.2%) times, and “Not effective” responses 94 (52.2%) and 84 (46.7%) times, respectively. No significant differences were found between the two LLMs in any category (*p* > 0.05).

**Table 3:** The prevalence of responses from GPT-4o and GPT-4 indicating the “Effective”, “Uncertain”, and “Not effective” for six diseases.

	Effectiveness	GPT-4o	GPT-4
Is [Supplement Name] effective against cancer?	Effective	1 (3.3%)	1 (3.3%)
	Uncertain	9 (30.0%)	12 (40.0%)
	Not effective	20 (66.7%)	17 (56.7%)
Is [Supplement Name] effective against diabetes?	Effective	3 (10.0%)	2 (6.7%)
	Uncertain	14 (46.7%)	18 (60.0%)
	Not effective	13 (43.3%)	10 (33.3%)
Is [Supplement Name] effective obesity?	Effective	2 (6.7%)	2 (6.7%)
	Uncertain	11 (36.7%)	20 (66.7%)
	Not effective	17 (56.7%)	8 (26.7%)
Is [Supplement Name] effective against constipation?	Effective	6 (20.0%)	6 (20.0%)
	Uncertain	10 (33.3%)	11 (36.7%)
	Not effective	14 (46.7%)	13 (43.3%)
Is [Supplement Name] effective against joint pain?	Effective	3 (10.0%)	3 (10.0%)
	Uncertain	11 (36.7%)	6 (20.0%)
	Not effective	14 (46.7%)	21 (70.0%)
Is [Supplement Name] effective against hypertension?	Effective	3 (10.0%)	6 (20.0%)
	Uncertain	13 (43.3%)	9 (30.0%)
	Not effective	14 (46.7%)	15 (50.0%)
Total answer	Effective	18 (10.0%)	20 (11.1%)
	Uncertain	68 (37.8%)	76 (42.2%)
	Not effective	94 (52.2%)	84 (46.7%)

For individual diseases, both GPT-4o and GPT-4 gave the fewest “Effective” responses for cancer (1 each, 3.3%) and the most for constipation (6 each, 20%). For other diseases, “Effective” responses accounted for about 10%.

The fewest “Not effective” responses were for diabetes and obesity—13 from GPT-4o (43.3%) and 10 from GPT-4 (33.3%). The most were for cancer—20 from GPT-4o (66.7%) and 17 from GPT-4 (56.7%). For the other diseases, about 50% of responses indicated no effectiveness.

### 3.3 Accuracy of GPT responses

**Fig. 2:**
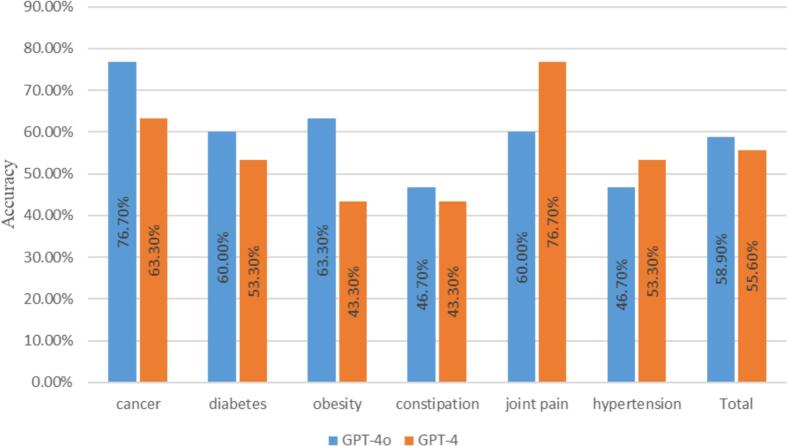
Accuracy of GPT-4o and GPT-4 across diseases.

The accuracy of the GPT responses was presented in Table 4 and Fig. 2. Overall, GPT-4o and GPT-4 had the accuracy of 58.9% and 55.6%. For individual diseases, two LLMs demonstrated the highest accuracy for cancer of 76.7% and 63.3%. The lowest accuracy was observed for constipation, with GPT-4o achieving 46.7% and GPT-4 achieving 43.3%. Significant difference was observed between the accuracy of cancer and constipation (*P* < 0.05).

**Table 4:** Correctness of response for different diseases.

	Correctness	GPT-4o	GPT-4
Is [Supplement Name] effective against cancer?	Correct n (%) [95% CI]	23 (76.7%) [59.1–88.2]	19 (63.3%) [45.5–78.1]
	Incorrect n (%) [95% CI]	7 (23.3%) [11.8–40.9]	11 (36.7%) [21.9–54.5]
Is [Supplement Name] effective against diabetes?	Correct n (%) [95% CI]	18 (60.0%) [42.3–75.4]	16 (53.3%) [36.1–69.8]
	Incorrect n (%) [95% CI]	12 (46.7%) [24.6–57.7]	14 (46.7%) [30.2–63.9]
Is [Supplement Name] effective against obesity?	Correct n (%) [95% CI]	19 (63.3%) [45.5–78.1]	13 (43.3%) [27.4–60.8]
	Incorrect n (%) [95% CI]	11 (36.7%) [21.9–54.5]	17 (56.7%) [39.2–72.6]
Is [Supplement Name] effective against constipation?	Correct n (%) [95% CI]	14 (46.7%) [30.2–63.9]	13 (43.3%) [27.4–60.8]
	Incorrect n (%) [95% CI]	16 (53.3%) [36.1–69.8]	17 (56.7%) [39.2–72.6]
Is [Supplement Name] effective against joint pain?	Correct n (%) [95% CI]	18 (60.0%) [42.3–75.4]	23 (76.7%) [59.1–88.2]
	Incorrect n (%) [95% CI]	12 (40.0%) [24.6–57.7]	7 (23.3%) [11.8–40.9]
Is [Supplement Name] effective against hypertension?	Correct n (%) [95% CI]	14 (46.7%) [30.2–63.9]	16 (53.3%) [36.1–69.8]
	Incorrect n (%) [95% CI]	16 (53.3%) [36.1–69.8]	14 (46.7%) [30.2–63.9]
Total	Correct n (%) [95% CI]	106 (58.9%) [51.6–65.8]	100 (55.6%) [48.3–62.6]
	Incorrect n (%) [95% CI]	74 (41.1%) [34.2–48.4]	80 (44.4%) [37.4–51.7]

A chi-square test showed no significant association between disease category and response correctness for either model (GPT-4o: χ^2^ = 4.27, df = 5, *p* = 0.51; GPT-4: χ^2^ = 5.13, df = 5, *p* = 0.40). Logistic regression analyses likewise indicated that none of the six disease categories significantly predicted accuracy (all p > 0.05). These results confirm that the observed accuracy differences among diseases were not statistically significant and likely reflect random variation rather than systematic performance differences.

### 3.4 Content analysis

Two authors (ML and XC) independently conducted open coding of all 360 GPT responses to identify recurring linguistic and structural patterns. Initial codes were generated inductively from the text rather than being predefined. The researchers then compared their coding results, discussed discrepancies, and refined the coding framework through iterative consensus meetings. Codes with conceptual similarity were aggregated into broader thematic categories, resulting in three primary themes: (1) templated structure of responses, (2) vague or ambiguous phrasing, and (3) omission of professional or medical guidance. This process ensured transparency and reliability in how the qualitative findings were developed from the raw data. The three themes of content analysis were shown in Table 5.

**Table 5:** Summary of qualitative themes identified in ChatGPT responses.

Theme	Description	n (%)
Templated structure of responses	Responses followed a fixed, formulaic pattern—typically starting with a general statement and listing effects, mechanisms, and cautions in similar order	360 (100%)
Ambiguous phrasing	Use of vague language such as “may help” or “possibly effective”	297 (82.5%)
Missing medical disclaimers	Absence of caution or recommendation to consult a doctor	24 (6.7%)

Overall, GPT-4 and GPT-4o responses followed a common template. The template was divided into three sections (Content Example 1). The first section provides a brief answer to the question of whether the supplement is effective against the disease. In the second section, the nutritional components of the supplements and their potential effects are explained in detail. The third section is a summary of the overall responses.

*Content Example 1 (Only English translated text was shown. The original text in Japanese was shown in Supplementary Material 4):*

*“(First Section)*

*Garlic may have some potential effects on obesity and weight management. Below are its possible effects:*

*(Second Section)*

*Boosting metabolism: Components in garlic may enhance metabolism, making it easier for the body to burn more calories.*

*Appetite suppression: The strong flavor of garlic may help reduce appetite, potentially preventing overeating and naturally lowering food intake.*

*Inhibiting fat accumulation: Some studies suggest that garlic might inhibit fat accumulation and help lower body fat percentage.*

*Supporting healthy weight management: Garlic contains many nutrients that support overall health and may be beneficial as part of a weight management plan.*

*(Third Section)*

*However, the effects of garlic on obesity and weight management heavily depend on individual lifestyle and overall diet. It's important not to rely solely on garlic but to maintain a balanced diet, regular exercise, and sufficient sleep as part of a healthy lifestyle. For diet and weight management, consulting with a nutritionist or physician is recommended. Garlic can play a supplementary role but should be considered as part of an overall diet strategy.”*

In the first section, 297 out of 360 responses included expressions indicating “possibility,” such as “there is a possibility of effectiveness” or “it may have some effects” (Content Example 2). In some cases, it emphasized that “there is no clear evidence of its effectiveness for the disease,” followed by “however, there is a possibility of effectiveness” (Content Example 3).

*Content Example 2:*

*“Garlic may have some potential effects on obesity and weight management.”*

*Content Example 3:*

*“Black vinegar contains antioxidants that are beneficial for general health maintenance, but it cannot be definitively stated that it has a direct effect on cancer. However, black vinegar may reduce the risk of cancer by neutralizing free radicals in the body and reducing cellular damage.”*

In the second section, even if the viewpoint of the question was correct, other incorrect claims were often included. For instance, in response to the question “Are DHA and EPA effective for diabetes?” the reply states that “there is no clear conclusion about the effect,” which is accurate. However, the response also claims that “DHA and EPA are effective in reducing cardiovascular risk,” which is incorrect because there is no evidence that DHA and EPA can effectively reduce cardiovascular risk (Content Example 4).

*Content Example 4:*

*“Various studies have examined the effects of DHA (docosahexaenoic acid) and EPA (eicosapentaenoic acid) on diabetes, but the results are sometimes contradictory, and no clear conclusions have been reached. Below are the potential effects of DHA and EPA on diabetes based on current scientific understanding:*

*Improved insulin sensitivity: Some studies suggest that omega-3 fatty acids may improve insulin sensitivity and reduce the risk of type 2 diabetes.*

*Reduction of inflammation: DHA and EPA have anti-inflammatory properties that may help alleviate chronic inflammation in diabetic patients.*

*Blood glucose control: Some studies indicate that omega-3 fatty acids may aid in blood glucose control, though other studies have not confirmed significant effects.*

*Reduction of cardiovascular risk: Diabetic patients are at higher risk for cardiovascular diseases, and DHA and EPA may support cardiovascular health and help reduce related risks.*

*Due to inconsistent research findings, a cautious approach is necessary when considering DHA and EPA as part of diabetes treatment. The optimal treatment plan for each individual should be determined in consultation with healthcare providers and based on personal health conditions and existing diabetes management strategies. Additionally, DHA and EPA intake should ideally be part of an overall healthy diet.”*

In the third section, 336/360 responses mentioned that consumers should follow the guidelines of doctors, nutritionists, medical professionals, healthcare providers, specialists, attending physicians, experts, and medical institutions (Content Example 5). However, in responses of GPT-4 to questions “Are green juice or calcium effective for joint pain?” and “Are blueberries effective for obesity?” and in responses GPT-4o to questions “Are black vinegar, collagen, soy isoflavones, DHA, garlic, vitamin C, placenta, blueberries, prunes, or calcium effective for obesity?”; “Are collagen, probiotics, garlic, blueberries, soy isoflavones, DHA, vitamin C, placenta, blueberries, or prunes effective for constipation?”; and “Are blueberries effective for cancer?” did not include a recommendation to follow such guidelines (Content Example 6).

*Content Example 5:*

*“Therefore, when using soy isoflavones for cancer treatment or prevention, it is essential to consult thoroughly with a doctor or specialist and receive appropriate advice.”*

*Content Example 6:*

*“Blueberries are believed to have antioxidant properties that may help in cancer prevention. Specifically, flavonoids such as anthocyanins in blueberries play a role in neutralizing free radicals in the body and protecting cells. This suggests they may reduce the risk of developing cancer.*

*However, there is no scientific evidence that eating blueberries can directly cure cancer. For cancer prevention or treatment, a balanced diet, regular exercise, and periodic health checkups are crucial. A healthy diet, including blueberries, may contribute to reducing cancer risk, but it cannot be claimed to treat cancer on its own.”*

## 4 Discussion and conclusion

### 4.1 Discussion

To our knowledge, this is the first study evaluating the accuracy and reliability of LLMs in the DS domain. Quantitative analysis showed that, out of 180 responses, GPT-4 and GPT-4o gave definitive “effective” answers only 18 (10.0%) and 20 (11.1%) times, respectively. About 40% of the responses were uncertain and 50% indicated no effect, suggesting a cautious and conservative stance.

For cancer, both LLMs gave only one “effective” response (3.3%), significantly fewer than for other diseases. This may be because cancer is a life-threatening illness, and DS products are rarely marketed as cancer treatments. A Japanese study on DS advertisements found no advertisements that claimed DS were effective against cancer [42], implying less misinformation in this area. With fewer misleading sources in the training data and more scrutiny in cancer-related content, LLMs are less likely to respond affirmatively. Additionally, authoritative organizations and medical literature emphasize the lack of evidence for DS in cancer treatment [39], [43], [44], reinforcing the LLMs' caution.

When comparing LLM outputs with evidence-based effectiveness, the overall accuracy was 58.9% for GPT-4o and 55.6% for GPT-4, with no significant difference. In contrast to previous studies showing ChatGPT's high accuracy in dietary planning and nutrition [10], [13], [14], [18], its performance in DS was less satisfactory. This may be due to two factors. First, nutrition and dietary planning are based on rigorously validated principles found in textbooks and guidelines, allowing LLMs to learn from high-quality, standardized data. In contrast, DS-related content often comes from ads, blogs, or user reviews, varying in scientific accuracy [24], [33]. Second, nutrition involves structured knowledge with clear guidelines and consensus-backed answers, aligning well with LLM strengths. However, DS effects remain controversial. For example, studies on soy isoflavones and diabetes show inconsistent outcomes even among postmenopausal women—some positive [45], others negative [46]. This lack of consensus creates noisy training data, making it harder for LLMs to offer accurate, reliable responses in the DS field.

Moreover, although GPT-4o is known to feature a more efficient architecture, faster response times, and enhanced reasoning and multimodal integration compared with GPT-4, and previous studies have reported that GPT-4o outperforms GPT-4 in structured medical knowledge tasks [7], our findings revealed no notable difference in accuracy or caution level between the two models in the dietary supplement domain. This may be explained by the fact that GPT-4o's architectural improvements primarily optimize inference efficiency and contextual coherence rather than domain-specific factual retrieval. In topics such as dietary supplements—where training data are limited, heterogeneous, and often commercially biased—both models likely rely on similar underlying language-generation and safety-alignment mechanisms that favor cautious or equivocal phrasing when evidence is uncertain. In other words, GPT-4o's improved reasoning efficiency does not directly translate into higher factual accuracy when the model must synthesize conflicting or low-quality evidence. These results imply that model evolution may enhance fluency and responsiveness but not necessarily factual reliability in complex health information contexts.

In addition to quantitative analysis, we conducted content analysis of LLMs' responses. GPT-4 and GPT-4o showed highly templated structures: an initial one-sentence answer, followed by explanation of DS components and effects, and ending with a summary. No notable differences in accuracy or style were found between the two LLMs.

In the first section, 297 of 360 responses included phrases like “possibly effective” or “there is a possibility,” contributing to around 40% of responses being categorized as “uncertain.” This resulted in ChatGPT providing “uncertain” responses in numerous cases where the evidence-based outcome was “not effective.” Our statistics show that GPT-4o produced 46 erroneous responses under this pattern, while GPT-4 produced 45, accounting for 62.2% and 56.3% of all incorrect answers respectively—far exceeding the proportion of errors from other patterns. Even when concluding “not effective,” the LLMs often added qualifiers like “there is a possibility of effectiveness.” Compared to structured exams, GPT-4 and GPT-4o tended to offer ambiguous rather than definitive responses in DS contexts. This may stem from a tendency toward “sycophancy,” which refers to the tendency of an LLM to produce overly agreeable or user-aligned answers, even when such responses deviate from factual accuracy. [47], [48]. Such ambiguity, especially in fields with unclear evidence, risks misleading users. Future updates should aim to reduce such vague expressions to minimize misinformation.

In the second section, GPT-4 and GPT-4o occasionally provided irrelevant and incorrect information. For instance, after stating in the first section that a DS has no effect on the disease in question, they may add information to the second section about an effect that does not exist. There are two possible reasons for this finding. Firstly, similar to the explanation above, LLMs may generate positive responses that contradict their original output to cater to user expectations. Secondly, prior research identified issues with LLMs, providing overly detailed and redundant responses [49], [50]. Consequently, LLMs may include additional content unrelated to a question. The controversial nature of many claims regarding the effects of DS can lead to the inclusion of incorrect and irrelevant information.

In the third section, GPT-4 and GPT-4o, in most cases, advise users to follow the guidance of doctors and other professionals, which is a correct and responsible suggestion. However, 6.7% of responses (24/360) failed to include this recommendation. Among the 24 responses that did not include this recommendation, 12 were correct, representing a 50% accuracy rate, which was not significantly different from the overall accuracy. However, when analyzing the disease distribution of these responses, we found that 12 were related to obesity, 10 to constipation, and only one each to cancer and joint pain. This finding is consistent with our previous conclusion that ChatGPT tends to provide more assertive and therefore higher-risk answers for non-life-threatening conditions such as obesity and constipation. We observed that omissions of this advice were more frequent in responses related to “obesity” and “constipation.” We believe that future updates should ensure that LLMs include the instructions to “follow your doctor's advice” in all health-related responses to prevent users from making decisions on their own.

From a health communication perspective, our findings highlight the double-edged role of LLMs such as ChatGPT in shaping public understanding of health information. AI-generated responses can simplify complex medical concepts and improve accessibility, supporting health literacy and informed choices. Yet, we found that ChatGPT often uses vague or overly agreeable language, which may create a “credibility illusion,” causing users to trust polished answers regardless of evidence quality. In areas like dietary supplements, where evidence is often weak and commercial influence strong, this can mislead consumers and widen information gaps, especially among vulnerable populations such as patients with chronic illnesses or individuals with low health literacy. Ethically, this raises concerns about harm from misinformation and underscores the need for safeguards such as transparent labeling, reference citation, and regulatory oversight to protect users. Embedding core health communication principles—audience empowerment, transparency, and informed choice—into AI system design is crucial to ensure these tools promote, rather than compromise, public health.

To mitigate the risk of misinformation, several practical strategies can be considered. First, prompting strategies can be optimized to reduce ambiguity and elicit more evidence-based responses—for example, by instructing LLMs to cite sources or to explicitly differentiate between confirmed and uncertain evidence. Second, model outputs can be aligned or cross-checked with authoritative dietary supplement databases (e.g., the NIH Office of Dietary Supplements, Japan's “Health Food Function Claims” database) to improve factual consistency. Third, embedding mandatory disclaimers at the end of each response, reminding users to consult healthcare professionals, could help prevent overreliance on AI-generated advice. Together, these strategies can enhance the safety, transparency, and reliability of LLM-assisted health communication regarding dietary supplements.

Finally, we developed a framework to evaluate the effects of DS on “cancer,” “diabetes,” “obesity,” “constipation,” “joint pain,” and “hypertension.” We look forward to further studies using our evaluation framework to assess the effectiveness of DS for various diseases.

#### 4.1.1 Limitations

First, this study evaluated the accuracy and reliability of GPT-4 and GPT-4o in the field of DS but did not explore the potential impact of their responses on users' behaviors and decisions. ChatGPT is widely used for health-related queries, and its responses may not only convey knowledge but also influence users' perceptions, trust, and purchasing behaviors. We could not assess whether inaccurate or ambiguous responses might lead users to rely on ineffective supplements or overlook more effective treatments. Future research should include user behavior simulation experiments, inviting participants from diverse backgrounds to read LLM responses and observe behavioral outcomes, such as purchasing DS, seeking professional advice, or changing health habits.

Second, although this study assessed GPT-4 and GPT-4o in a Japanese-language context, it did not evaluate their accuracy across other languages or cultures. Given their multilingual design, LLM performance may vary by language characteristics, cultural norms, and training data distribution. English, as the primary training language, benefits from more extensive authoritative sources, while languages like Japanese, Chinese, and Arabic may be underrepresented, potentially impacting response accuracy.

Third, because we collected only one response per question, our results reflect single-shot, real-world use and may be influenced by stochastic variability. We did not control model temperature or sampling parameters, so variability (including occasional hallucinations) is part of the outcome. Future research could compare results using repeated sampling or deterministic decoding to measure stability.

Fourth, this study is focused solely on OpenAI's GPT-4 and GPT-4o. At the time of data collection (September 2024), GPT-4o was widely recognized as the strongest publicly available general-purpose LLM according to major benchmark evaluations such as MMLU and MedMCQA. Our research objective was to replicate realistic user scenarios in which ordinary Japanese consumers query widely accessible generative AI tools about dietary supplements. For this reason, we did not include closed or medically fine-tuned models such as MeLLama or GatorTronGPT. Future research should expand benchmarking to include other strong general models and medically fine-tuned LLMs to evaluate whether domain-specific optimization leads to higher factual accuracy or different risk profiles in supplement dietary field.

#### 4.1.2 Innovation

This study introduces several key innovations in the evaluation of LLMs in the field of health information. To our knowledge, this is the first research to systematically assess both the accuracy and reliability of GPT-4 and GPT-4o responses concerning DS, a domain characterized by widespread misinformation and scientific controversy. While previous studies have explored LLM performance in structured medical exams or general nutrition advice, none have focused on DS, which often lacks scientific consensus and is vulnerable to commercial exploitation.

Our innovation lies not only in the domain of study but also in methodological rigor. We employed a mixed-method approach combining quantitative accuracy evaluation with qualitative content analysis, allowing us to uncover not just what LLMs say about DS, but how they say it—revealing patterns such as ambiguous phrasing, sycophantic tendencies, and omission of critical disclaimers. This dual-layered method offers a new standard for LLM evaluation in health contexts.

Importantly, we also developed a novel evidence-based evaluation framework to assess the effectiveness of 30 popular DS across six major diseases. This framework synthesizes experimental findings and expert clinical judgment to provide a standardized benchmark, enabling future studies to assess LLM performance consistently across DS-related topics.

Given the rapid integration of generative AI into health information-seeking behaviors, evaluating LLMs in controversial, high-uncertainty fields like DS is urgently needed. Our approach offers a transferable blueprint for assessing AI reliability in other complex domains, and our findings contribute to the growing discourse on responsible AI deployment in public health. We believe this study will support the development of safer, more accurate AI tools and guide developers, healthcare professionals, and policymakers alike.

### 4.2 Conclusion

This is the first study to evaluate the accuracy and reliability of LLM responses to DS. GPT-4 and GPT-4o showed no major differences and generally took a conservative stance, with only 10% affirming DS effectiveness. Cancer received the fewest such affirmations. However, ambiguous and occasionally inaccurate information suggests that LLMs may try to align with user expectations, posing misinformation risks. We recommend LLMs consistently advise users to follow professional medical guidance. The accuracy of DS responses was around 57%, lower than in prior LLM studies on nutrition, likely due to the scientific ambiguity surrounding DS. Lastly, we developed a framework for evaluating DS effectiveness across six diseases, which we hope future research will adopt.

## CRediT authorship contribution statement

**Mingxin Liu:** Writing – review & editing, Writing – original draft, Validation, Software, Methodology, Funding acquisition, Data curation, Conceptualization. **Tsuyoshi Okuhara:** Writing – review & editing, Writing – original draft, Supervision, Methodology, Conceptualization. **Ritsuko Shirabe:** Writing – review & editing, Methodology, Data curation. **Yuriko Nishiie:** Writing – review & editing, Methodology, Data curation. **Xinyi Chang:** Validation, Data curation. **Hiroko Okada:** Writing – review & editing, Software, Methodology. **Takahiro Kiuchi:** Writing – review & editing, Resources.

## Declaration of competing interest

The authors declare the following financial interests/personal relationships which may be considered as potential competing interests:

Mingxin Liu reports financial support was provided by 10.13039/501100001691Japan Society for the Promotion of Science. If there are other authors, they declare that they have no known competing financial interests or personal relationships that could have appeared to influence the work reported in this paper.

## Appendix A Supplementary data

Supplementary material 1 Image 1

Supplementary material 2 Image 2

Supplementary material 3 Image 3

Supplementary material 4 Image 4
