# Supplementary material for: Validity and reliability of ChatGPT's responses on dietary supplements in Japan: A quality assessment and content analysis
Source: PEC Innov. 2026 Feb 21;8:100461. doi: 10.1016/j.pecinn.2026.100461 (PMC12955162; doi:10.1016/j.pecinn.2026.100461)
Supplement: Supplementary file 1 — Supplementary material 1 [file mmc1.docx]

**Title page**

**Large Language Models Show Moderate Accuracy and High Ambiguity in Responses About Dietary Supplements in Japan: A Quality Assessment and Content Analysis**

Mingxin Liu ^1^, MA, E-mail: liumingxin98@g.ecc.u-tokyo.ac.jp

Tsuyoshi Okuhara ^2^, PhD, E-mail: [okuhara-ctr@umin.ac.jp](mailto:okuhara.hc@gmail.com)

Ritsuko Shirabe ^3^, PhD, E-mail: rshirabe-tky@umin.ac.jp

Yuriko Nishiie ^1^, MPH, E-mail: yuriko.nishiie.728@gmail.com

Hiroko Okada ^2^, PhD, E-mail: [okadahiroko-tky@umin.ac.jp](mailto:okadahiroko-tky@umin.ac.jp)

Takahiro Kiuchi ^2^, PhD, E-mail: tak-kiuchi@umin.ac.jp

1. Department of Health Communication, Graduate School of Medicine, The University of Tokyo, Tokyo, Japan.

2. Department of Health Communication, School of Public Health, Graduate School of Medicine, The University of Tokyo, Tokyo, Japan.

3. University hospital Medical Information Network (UMIN) Center, The University of Tokyo Hospital, Tokyo, Japan

Correspondence should be addressed to Mingxin Liu, Department of Health Communication, Graduate School of Medicine, The University of Tokyo, Hongo 7-3-1, Bunkyo, Tokyo, Japan; telephone: 81-90-6242-1998; e-mail: liumingxin98@g.ecc.u-tokyo.ac.jp.

**Supplementary Materials 1. An example of effectiveness reports dietary supplements for various body system published by National Institute of Health and Nutrition in Japan.**

**Translated version in English.**

| **Item** | **Information** |
| --- | --- |
| **Name** | Agaricus, Himematsutake, Mematsutake, Kawariharatake, Agaricus blazei  [English] Agaricus [Scientific Name] Agaricus blazei Murill |
| **Overview** | Agaricus is a type of mushroom that grows from the ground, featuring a long, thick stem and a strong aroma. It is also known as Agaricus blazei or Himematsutake. Since its introduction from Brazil in 1965, it has been cultivated artificially. However, its characteristics and components vary depending on the strain, cultivation conditions, and production region. |
| **Regulations & Legal Classification** | - Food and Drug Classification:   - Fruiting body: It is classified as a "natural ingredient (raw material) that is not considered a pharmaceutical product unless it claims medicinal effects." |
| **Characteristics & Quality of Components** | |
| **Main Components & Properties** | - Compared to other mushrooms, Agaricus contains a higher amount of crude protein. - It has been reported to contain polysaccharides, vitamins B1, B2, niacin, B6, biotin, pantothenic acid, folic acid, amino acids, vitamin D, magnesium, and potassium (PMID: 18604247). - The β-glucan content in Himematsutake extracts and commercial Agaricus products varies among products (2005215401). - The agaritine content in Himematsutake has been reported to range from 500 to 5,000 mg/kg (dry weight) (PMID: 19680875). - Some Agaricus products have been found to contain high levels of cadmium, but voluntary standards have been implemented to address this issue (104). - Due to the significant variation in quality among Agaricus-containing products, certain voluntary guidelines require overdose testing in humans (PMID: 18408335). |
| **Analysis Methods** | - Agaricus, like other mushroom and yeast products, contains β-D-glucan, but the structural characteristics and molecular weight distribution differ significantly depending on the mushroom type. There is no consensus on the relationship between structure and activity. The total β-glucan content in mushrooms has been measured using a specific detection kit (2005215401). - Ergosterol, a vitamin D precursor found in mushrooms, has been analyzed using gas chromatography-mass spectrometry (GC-MS) (PMID: 9872771). - The analysis of agaritine and its derivatives has been conducted using HPLC-fluorescence detection (PMID: 16755074) and LC-electrospray ionization tandem mass spectrometry (PMID: 17071520). |
| **Efficacy & Clinical Evaluation in Humans** | |
| **Cardiovascular & Respiratory System** | - Randomized Controlled Trial (RCT)   - A double-blind, randomized, placebo-controlled trial conducted in Japan on 33 individuals with mild hypertension examined the effects of consuming Agaricus granules (2g, twice a day, n=10, mean age: 49.3±10.7 years) or Agaricus extract (50 mL, twice a day, n=12, mean age: 47.4±9.0 years) for four weeks. No significant changes were observed in systolic/diastolic blood pressure, pulse rate, body weight, BMI, or biochemical markers (2007007822). |
| **Digestive System & Liver** | No relevant studies were found in the reviewed literature. |
| **Diabetes & Endocrine System** | - RCT   - A double-blind, randomized, placebo-controlled trial conducted in China involved 60 participants with type 2 diabetes who had been on Gliclazide and Metformin for over six months. The trial examined the effects of Agaricus extract (1,500 mg/day, content unspecified) for 12 weeks. A significant reduction in the HOMA-IR marker (insulin resistance index) was observed, but no effects on fasting blood glucose, HbA1c, insulin, or adiponectin levels were found (PMID: 17309383). |
| **Reproductive & Urinary System** | No relevant studies were found in the reviewed literature. |
| **Neurological & Sensory System** | No relevant studies were found in the reviewed literature. |
| **Immune System, Cancer & Inflammation** | - RCT   - A double-blind, crossover, placebo-controlled trial in Japan investigated the effects of Agaricus (A. brasiliensis KA21) at 3 g/day for 7 days in eight healthy adults (mean age: 22.3±0.5 years). Results showed a significant increase in NK (natural killer) cell activity against K562 cells, but no change in NK cell count (PMID: 18604247).   - Another double-blind, randomized, placebo-controlled trial in Brazil involved 57 healthy elderly women (test group: 28 participants, mean age: 70.4±6.3 years). Participants consumed Agaricus dried extract (300 mg, three times a day) for 60 days. No significant effects were observed on BMI, waist circumference, blood pressure, or cytokine levels (IL-6, IFN-γ, TNF-α) (PMID: 22010847). |
| **Skeletal & Muscular System** | No relevant studies were found in the reviewed literature. |
| **Growth & Development** | No relevant studies were found in the reviewed literature. |
| **Obesity** | No relevant studies were found in the reviewed literature. |
| **Other Conditions** | No relevant studies were found in the reviewed literature. |
| **References** | |
| **(2005215401) 東京都立衛生研究所研究年報.2002;53:165-8.**  **(PMID:12204626) Carbohydr Res. 2002 Sep 3;337(15):1417-21.**  **(PMID:9872771) Appl Environ Microbiol. 1999 Jan;65(1):138-42.**  **(104) 「担子菌類中の有害物質の評価に関する研究」（平成１６年度厚生労働科学 研究報告書）**  **(PMID:15833380) Food Chem Toxicol. 2005 Jul;43(7):1047-53.**  **(PMID:17105737) Jpn J Clin Oncol. 2006 Dec;36(12):808-10.**  **(2005013083) 老年消化器病. 2004;16（2）:101-7.**  **(2004276900) 皮膚病診療. 2004;26（8）:967-70.**  **(2004252918) Minophagen Med Rev. 2004;49(3):176-8.**  **(2004149979) 肝臓. 2004;45(2):96-108. (2004125341) 日本胸部臨床. 2003;62(11):1027-31. (2005001969) 肺癌. 2004;44(3):167-71.**  **(PMID:17309383) J Altern Complement Med. 2007 Jan-Feb;13(1):97-102.**  **(2006138431) プラクティス. 2006;23(1):89-92.**  **(2006113539) 臨床神経学. 2006;46(1):67.**  **(2006035857) 臨床皮膚科. 2005;59(10):1013-5.**  **(2006209905) 皮膚の科学. 2006;5(1):102.**  **(PMID:16755074) Chem Pharm Bull (Tokyo). 2006 Jun;54(6):922-4.**  **(PMID:17071520) Food Addit Contam. 2006 Nov;23(11):1179-86.**  **(PMID:7737599) Food Chem Toxicol. 1995 Apr;33(4):257-64.**  **(PMID:9276636) Carcinogenesis. 1997 Aug;18(8):1603-8.**  **(PMID:17707568) Food Chem Toxicol. 2008 Jan;46(1):87-95.**  **(PMID:9571772) Biosci Biotechnol Biochem. 1998 Mar;62(3):434-7**  **(PMID:18154438) JARMAM 臨床微生物迅速診断研究会誌.2007;18(2):103-7. (PMID:18353517) Food Chem Toxicol. 2008 Jun;46(6):1949-59.**  **(PMID:19170155) Phytother Res. 2009 Jul;23(7):906-12.**  **(PMID:19879310) Food Chem Toxicol. 2010 Jan;48(1):402-8.**  **(PMID:20649730) J Dermatol. 2010 Aug;37(8):773-5.**  **(PMID:22010847) Scand J Immunol. 2012 Mar;75(3):336-41. (2010342411) 糖尿病. 2010;53(8):647. (2010017845) 西日本泌尿器科. 2009;71:144.**  **(PMID:19680875) Food Addit Contam Part A Chem Anal Control Expo Risk A ssess. 2009 Jan;26(1):82-93.**  **(PMID:9625538) Cancer Immunol Immunother. 1998 May;46(3):147-59.**  **(PMID:12224654) Biosci Biotechnol Biochem. 2002 Jul;66(7):1610-4.**  **(22) メディカルハーブ安全性ハンドブック第2版東京堂出版林真一郎ら監訳**  **(2014145301) Clinical Journal of Gastroenterology. 2013; 6(2): 139-44.**  **(91) Registry of Toxic Effects of Chemical Substances (RTECS) (2007007822) Health Sci. 2006;22(3):316-28.**  **(2012341380) J Environ Dermatol Cutan Allergol. 2012;6(3):277.**  **(2007343698) 日本腎臓学会誌. 2007;49(6):594.**  **(PMID:18604247) Evid Based Complement Alternat Med. 2008 J un;5(2):205-19. (PMID:18408335) 日本薬理学雑誌.2008 Apr;131(4):252-7.**  **(2005052247) 日本癌治療学会誌. 2004:39(2);426.**  **(2018108228) 日本呼吸器学会誌. 2017:6(3);186-9.**  **(30) 「医薬品の範囲に関する基準」(別添1、別添2、一部改正について)** | |

**
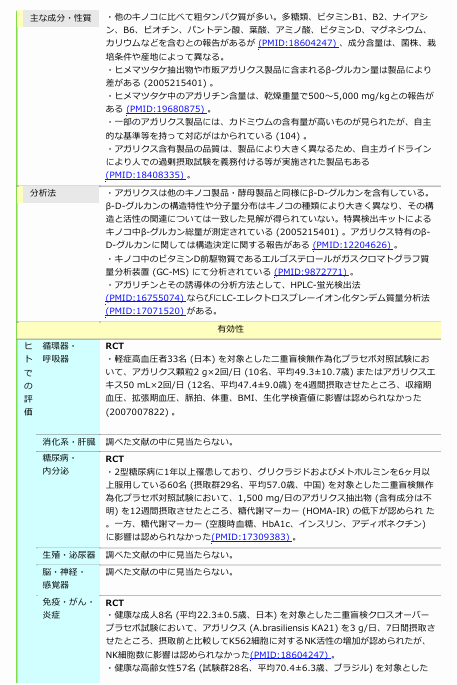

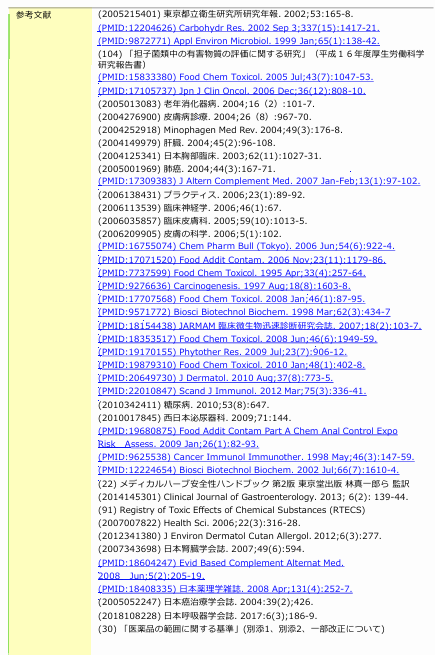

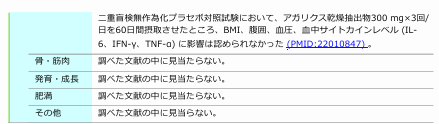

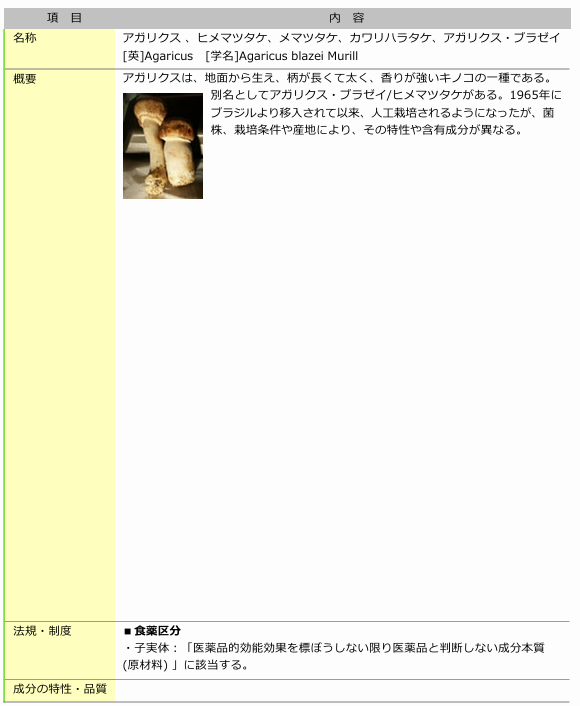
Original version in Japanese.**

**Supplementary materials 2. Evaluation framework developed by two medical professionals for evidence-based effects.**

|  | Effective | Uncertain | Not effective |
| --- | --- | --- | --- |
| Cancer | For cancer patients: 1. Extension of overall survival time 2. Disease-free survival time 3. Symptom improvement 4. Objective response rate 5. Progression-free survival time For healthy adults: 1. Reduction in incidence/mortality risk as shown in meta-analyses, RCTs, or prospective cohort studies. | 1. Effects at the vitro test level (e.g., T-cell, NK-cell studies). 2. Effects observed in animal experiments. 3. Reduction in incidence/mortality risk observed in studies other than meta-analyses, RCTs, or prospective cohort studies (e.g., case-control studies). 4. Theoretical references to potential effects (e.g., reduction of oxidative stress, anti-inflammatory effects). 5. Simultaneous reports of positive and negative results. | 1. No research exists, and the effect is considered unlikely, even theoretically. 2. Comparative trials on direct effect indicators concluded no effect. |
| Diabetes | 1. Improvement in fasting blood glucose levels. 2. Improvement in HbA1c. 3. Improvement in insulin resistance markers (e.g., HOMR-IR × fasting insulin levels). 4. Improvement in insulin secretion indicators (e.g., 75g OGTT, blood insulin/C-peptide levels post-meal, daily C-peptide levels in urine). 5. Pancreatic β-cell function evaluation indicators (e.g., HOMA-β, proinsulin/insulin ratio, proinsulin/C-peptide ratio). 6. Physical measurements (e.g., BMI, blood pressure, waist circumference). 7. Improvement in complications (e.g., nephropathy, retinopathy, neuropathy, atherosclerosis indicators). 8. Improved perinatal outcomes in gestational diabetes (e.g., reduced fetal death, large babies, cesarean sections, shoulder dystocia, neonatal respiratory distress syndrome) and decreased risk of postpartum diabetes onset. 9. Reduction in incidence/mortality risk based on meta-analyses, RCTs, or prospective cohort studies (for healthy adults). | 1. Effects observed in vitro testing. 2. Effects observed in animal studies. 3. Theoretical references to potential effects (e.g., anti-inflammatory effects improving insulin resistance). 4. Simultaneous reports of positive and negative results. 5. Reduction in incidence/mortality risk observed in studies other than meta-analyses, RCTs, or prospective cohort studies (e.g., case-control studies, for healthy adults). | 1. No research exists, and the effect is considered unlikely, even theoretically. 2. Comparative trials on direct effect indicators concluded no effect. |
| Obesity | 1. Weight reduction and BMI improvement. 2. Decrease in visceral fat and waist circumference. 3. Improvement in obesity-related health disorders (e.g., diabetes, hypertension). | 1. Effects observed in vitro testing. 2. Effects observed in animal studies. 3. Theoretical references to potential effects. 4. Simultaneous reports of positive and negative results. | 1. No research exists, and the effect is considered unlikely, even theoretically. 2. Comparative trials on direct effect indicators concluded no effect. |
| Constipation | 1. Reduction in straining during defecation. 2. Improvement in stool consistency (BSFS). 3. Improvement in residual stool sensation. 4. Improvement in defecation difficulty. 5. Reduction in manual defecation assistance. 6. Increase in spontaneous bowel movements. | 1. Effects observed in vitro testing. 2. Effects observed in animal studies. 3. Theoretical references to potential effects (e.g., increased bile secretion, regulation of intestinal immunity). 4. Simultaneous reports of positive and negative results. | 1. No research exists, and the effect is considered unlikely, even theoretically. 2. Comparative trials on direct effect indicators concluded no effect. |
| Joint pain | 1. Pain improvement. | 1. Effects observed in vitro testing. 2. Effects observed in animal studies. 3. Theoretical references to potential effects. 4. Simultaneous reports of positive and negative results. | 1. No research exists, and the effect is considered unlikely, even theoretically. 2. Comparative trials on direct effect indicators concluded no effect. |
| Hypertension | 1. Reduction in systolic blood pressure. 2. Reduction in diastolic blood pressure. | 1. Effects observed in vitro testing. 2. Effects observed in animal studies. 3. Theoretical references to potential effects. 4. Simultaneous reports of positive and negative results. | 1. No research exists, and the effect is considered unlikely, even theoretically. 2. Comparative trials on direct effect indicators concluded no effect. |

**Supplementary materials 3. The guideline developed by two medical professionals used to evaluate the effectiveness of dietary supplements for specific diseases as presented in the GPT responses**

| **Category** | **Definition** | **Examples** |
| --- | --- | --- |
| **A** | The text consists of statements affirming the direct or indirect effects of the supplement. No mention of denying effectiveness.  - | - Some studies show effectiveness.  - There are indirect effects.  - There are still few studies demonstrating effectiveness, but it is promising. |
| **B** | The text consists of ambiguous statements, such as both positive and negative statements about the effect, or insufficient scientific evidence for the effect, although the effect is positive. | - Mentions both effectiveness and lack of effectiveness.  - Acknowledges indirect effects but questions direct effects.  - There is some scientific evidence of effectiveness, but it is insufficient or not fully established.  - There are individual differences.  - The independent effect is limited, but when combined with treatments, it may be effective (supplementary effect). |
| **C** | The text consists of statements that question or do not mention the effect on the disease. | - It is not effective, but it is important for health.  - Effectiveness is questioned.  - Scientific evidence of effectiveness is limited.  - It does not have an effect on constipation, but it may improve gut health.  - There is little evidence that it relieves joint pain, but it helps maintain joint health. |

**Supplementary Materials 4. Evidence-based effects of 30 Dietary supplements for six diseases.**

| DS name | Cancer | Diabetes | Obesity | Constipation | Joint pain | Hypertension |
| --- | --- | --- | --- | --- | --- | --- |
| Aojiru | C | C | C | C | C | C |
| Agaricus | B | B | C | C | C | C |
| Ginkgo Biloba | C | C | C | C | C | B |
| Turmeric | C | C | C | C | A | C |
| Ornithine | C | C | B | C | C | C |
| Oyster Extract | C | C | C | C | C | C |
| Chitin and Chitosan | C | C | B | C | C | B |
| Glucosamine | B | C | C | C | B | C |
| Black Vinegar | C | C | C | C | C | A |
| Chlorella | B | C | C | C | C | C |
| Korean Ginseng | B | B | C | C | C | C |
| Coenzyme Q10 | B | C | C | C | C | B |
| Collagen | C | C | C | C | B | C |
| Squalene | C | C | C | C | C | C |
| Chinese softshell turtle | C | C | C | C | C | C |
| Soy Isoflavone | B | B | B | C | C | B |
| DHA & EHA | B | B | B | C | C | B |
| Lactic acid bacteria | C | C | B | C | C | C |
| Garlic | B | B | C | C | B | B |
| Hyaluronic acid | C | C | C | C | A | C |
| Vitamin E | B | C | C | C | C | C |
| Vitamin C | B | C | C | C | C | B |
| Placenta | C | C | C | C | C | C |
| Blueberry & Bilberry | C | B | C | C | C | C |
| Prune | C | C | C | C | C | C |
| Propolis | C | B | C | C | C | C |
| Maca | C | C | C | C | C | C |
| Euglena | C | C | C | C | C | C |
| Royal jelly | C | B | C | C | C | B |
| Calcium | B | B | B | C | C | C |

**Supplementary materials 5. Examples of original text in Japanese included in content analysis.**

*Content Example 1 (Translated text in English):*

*"(First Section)*

*Garlic may have some potential effects on obesity and weight management. Below are its possible effects:*

*(Second Section)*

*Boosting metabolism: Components in garlic may enhance metabolism, making it easier for the body to burn more calories.*

*Appetite suppression: The strong flavor of garlic may help reduce appetite, potentially preventing overeating and naturally lowering food intake.*

*Inhibiting fat accumulation: Some studies suggest that garlic might inhibit fat accumulation and help lower body fat percentage.*

*Supporting healthy weight management: Garlic contains many nutrients that support overall health and may be beneficial as part of a weight management plan.*

*(Third Section)*

*However, the effects of garlic on obesity and weight management heavily depend on individual lifestyle and overall diet. It's important not to rely solely on garlic but to maintain a balanced diet, regular exercise, and sufficient sleep as part of a healthy lifestyle. For diet and weight management, consulting with a nutritionist or physician is recommended. Garlic can play a supplementary role but should be considered as part of an overall diet strategy."*

*Content Example 1 (Original text in Japanese):*

*“（第一セクション）*

*ニンニクが肥満やダイエットに対してある程度の効果を持つ可能性があります。以下に、ニンニクの可能な効果を挙げます：*

*（第二セクション）*

*代謝の促進：ニンニクに含まれる成分が代謝を高める効果を持つ可能性があります。代謝が活発になると、体はより多くのカロリーを消費しやすくなります。*

*食欲抑制：ニンニクの強い風味が食欲を減少させる効果をもたらす場合があります。これにより、過食を防ぎ、食事の量を自然に減らすことができるかもしれません。*

*脂肪の蓄積抑制：一部の研究では、ニンニクが脂肪の蓄積を抑制し、体脂肪率の低下を助ける可能性が示唆されています。*

*（第三セクション）*

*健康的な体重管理のサポート：ニンニクは全体的な健康をサポートする多くの栄養素を含んでおり、健康的な体重管理プランの一部として役立つかもしれません。*

*しかし、肥満やダイエットに対するニンニクの効果は、個人の生活習慣や全体的な食事に大きく依存します。また、ニンニクだけに頼るのではなく、バランスの取れた食事、定期的な運動、十分な睡眠など、健康的なライフスタイルを維持することが重要です。ダイエットや体重管理に関しては、栄養士や医師と相談することをお勧めします。ニンニクは補助的な役割を果たすことはできますが、全体的なダイエット戦略の一部として捉えるべきです。”*

*Content Example 2 (Translated text in English):*

*"Garlic may have some potential effects on obesity and weight management."*

*Content Example 2 (Original text in Japanese):*

*“ニンニクが肥満やダイエットに対してある程度の効果を持つ可能性があります。”*

*Content Example 3 (Translated text in English):*

*" Black vinegar contains antioxidants that are beneficial for general health maintenance, but it cannot be definitively stated that it has a direct effect on cancer. However, black vinegar may reduce the risk of cancer by neutralizing free radicals in the body and reducing cellular damage."*

*Content Example 3 (Original text in Japanese):*

*“黒酢には抗酸化物質が含まれており、一般的な健康の維持に有益ですが、がんに対して直接的な効果があると断言することはできません。黒酢は体内の自由基を中和し、細胞の損傷を減らすことで、がんのリスクを低減する可能性があります。”*

*Content Example 4 (Translated text in English):*

*“Various studies have examined the effects of DHA (docosahexaenoic acid) and EPA (eicosapentaenoic acid) on diabetes, but the results are sometimes contradictory, and no clear conclusions have been reached. Below are the potential effects of DHA and EPA on diabetes based on current scientific understanding:*

*Improved insulin sensitivity: Some studies suggest that omega-3 fatty acids may improve insulin sensitivity and reduce the risk of type 2 diabetes.*

*Reduction of inflammation: DHA and EPA have anti-inflammatory properties that may help alleviate chronic inflammation in diabetic patients.*

*Blood glucose control: Some studies indicate that omega-3 fatty acids may aid in blood glucose control, though other studies have not confirmed significant effects.*

*Reduction of cardiovascular risk: Diabetic patients are at higher risk for cardiovascular diseases, and DHA and EPA may support cardiovascular health and help reduce related risks.*

*Due to inconsistent research findings, a cautious approach is necessary when considering DHA and EPA as part of diabetes treatment. The optimal treatment plan for each individual should be determined in consultation with healthcare providers and based on personal health conditions and existing diabetes management strategies. Additionally, DHA and EPA intake should ideally be part of an overall healthy diet.”*

*Content Example 4 (Original text in Japanese):*

*“DHA（ドコサヘキサエン酸）とEPA（エイコサペンタエン酸）が糖尿病に及ぼす影響については、複数の研究が行われていますが、その結果は一部で矛盾している場合もあり、明確な結論には至っていません。以下は、現在の科学的理解に基づくDHAとEPAが糖尿病に及ぼす可能性のある影響です。*

*インスリン感受性の改善: 一部の研究では、オメガ3脂肪酸がインスリン感受性を改善し、2型糖尿病のリスクを減少させる可能性が示唆されています。*

*炎症の低減: DHAとEPAには抗炎症作用があり、糖尿病患者における慢性的な炎症状態の軽減に役立つ可能性があります。*

*血糖コントロール: 一部の研究では、オメガ3脂肪酸が血糖コントロールに役立つ可能性があるとされていますが、他の研究では明確な効果が確認されていません。*

*心血管リスクの低減: 糖尿病患者は心血管疾患のリスクが高いですが、DHAとEPAは心血管系の健康をサポートし、これに関連するリスクを減少させる助けになる可能性があります。*

*これらの効果については、研究結果が一貫していないため、DHAとEPAを糖尿病治療の一環として用いる際には慎重なアプローチが必要です。個々の患者にとって最適な治療法は、医療提供者と相談し、個人の健康状態や既存の糖尿病管理計画に基づいて決定することが重要です。また、DHAとEPAの摂取は全体的な健康的な食生活の一環として行うことが望ましいです。”*

*Content Example 5 (Translated text in English, mentioning the need to follow professional guidance):*

*"Therefore, when using soy isoflavones for cancer treatment or prevention, it is essential to consult thoroughly with a doctor or specialist and receive appropriate advice."*

*Content Example 5 (Original text in Japanese, mentioning the need to follow professional guidance):*

*“したがって、大豆イソフラボンをがん治療や予防の目的で使用する場合は、医師や専門家と十分に相談し、適切なアドバイスを受けることが重要です。”*

*Content Example 6 (Translated text in English, not mentioning the need to follow professional guidance):*

*"Blueberries are believed to have antioxidant properties that may help in cancer prevention. Specifically, flavonoids such as anthocyanins in blueberries play a role in neutralizing free radicals in the body and protecting cells. This suggests they may reduce the risk of developing cancer.*

*However, there is no scientific evidence that eating blueberries can directly cure cancer. For cancer prevention or treatment, a balanced diet, regular exercise, and periodic health checkups are crucial. A healthy diet, including blueberries, may contribute to reducing cancer risk, but it cannot be claimed to treat cancer on its own."*

*Content Example 6 (Original text in Japanese, not mentioning the need to follow professional guidance):*

“*ブルーベリーには抗酸化作用があるため、がん予防に役立つ可能性があるとされています。特に、ブルーベリーに含まれるアントシアニンなどのフラボノイドは、体内のフリーラジカルを中和し、細胞を保護する役割があります。これにより、がんの発生リスクを下げる可能性が示唆されています。*

*しかし、ブルーベリーを食べることで直接がんを治す効果があるという科学的な証拠はありません。がんの予防や治療には、栄養バランスの取れた食事、適度な運動、定期的な健康診断が重要です。ブルーベリーを含めた健康的な食生活は、がんのリスクを減らす一助になるかもしれませんが、単体でがんに効くとは言えません。*”
