# Supplementary material for: Validity and reliability of ChatGPT's responses on dietary supplements in Japan: A quality assessment and content analysis
Source: PEC Innov. 2026 Feb 21;8:100461. doi: 10.1016/j.pecinn.2026.100461 (PMC12955162; doi:10.1016/j.pecinn.2026.100461)
Supplement: Supplementary file 2 — Supplementary material 2 [file mmc2.docx]

**Supplementary materials 2. Evaluation framework for evidence-based effects.**

|  | Effective | Uncertain | Not effective |
| --- | --- | --- | --- |
| Cancer | For cancer patients: 1. Extension of overall survival time 2. Disease-free survival time 3. Symptom improvement 4. Objective response rate 5. Progression-free survival time For healthy adults: 1. Reduction in incidence/mortality risk as shown in meta-analyses, RCTs, or prospective cohort studies. | 1. Effects at the vitro test level (e.g., T-cell, NK-cell studies). 2. Effects observed in animal experiments. 3. Reduction in incidence/mortality risk observed in studies other than meta-analyses, RCTs, or prospective cohort studies (e.g., case-control studies). 4. Theoretical references to potential effects (e.g., reduction of oxidative stress, anti-inflammatory effects). 5. Simultaneous reports of positive and negative results. | 1. No research exists, and the effect is considered unlikely, even theoretically. 2. Comparative trials on direct effect indicators concluded no effect. |
| Diabetes | 1. Improvement in fasting blood glucose levels. 2. Improvement in HbA1c. 3. Improvement in insulin resistance markers (e.g., HOMR-IR × fasting insulin levels). 4. Improvement in insulin secretion indicators (e.g., 75g OGTT, blood insulin/C-peptide levels post-meal, daily C-peptide levels in urine). 5. Pancreatic β-cell function evaluation indicators (e.g., HOMA-β, proinsulin/insulin ratio, proinsulin/C-peptide ratio). 6. Physical measurements (e.g., BMI, blood pressure, waist circumference). 7. Improvement in complications (e.g., nephropathy, retinopathy, neuropathy, atherosclerosis indicators). 8. Improved perinatal outcomes in gestational diabetes (e.g., reduced fetal death, large babies, cesarean sections, shoulder dystocia, neonatal respiratory distress syndrome) and decreased risk of postpartum diabetes onset. 9. Reduction in incidence/mortality risk based on meta-analyses, RCTs, or prospective cohort studies (for healthy adults). | 1. Effects observed in vitro testing. 2. Effects observed in animal studies. 3. Theoretical references to potential effects (e.g., anti-inflammatory effects improving insulin resistance). 4. Simultaneous reports of positive and negative results. 5. Reduction in incidence/mortality risk observed in studies other than meta-analyses, RCTs, or prospective cohort studies (e.g., case-control studies, for healthy adults). | 1. No research exists, and the effect is considered unlikely, even theoretically. 2. Comparative trials on direct effect indicators concluded no effect. |
| Obesity | 1. Weight reduction and BMI improvement. 2. Decrease in visceral fat and waist circumference. 3. Improvement in obesity-related health disorders (e.g., diabetes, hypertension). | 1. Effects observed in vitro testing. 2. Effects observed in animal studies. 3. Theoretical references to potential effects. 4. Simultaneous reports of positive and negative results. | 1. No research exists, and the effect is considered unlikely, even theoretically. 2. Comparative trials on direct effect indicators concluded no effect. |
| Constipation | 1. Reduction in straining during defecation. 2. Improvement in stool consistency (BSFS). 3. Improvement in residual stool sensation. 4. Improvement in defecation difficulty. 5. Reduction in manual defecation assistance. 6. Increase in spontaneous bowel movements. | 1. Effects observed in vitro testing. 2. Effects observed in animal studies. 3. Theoretical references to potential effects (e.g., increased bile secretion, regulation of intestinal immunity). 4. Simultaneous reports of positive and negative results. | 1. No research exists, and the effect is considered unlikely, even theoretically. 2. Comparative trials on direct effect indicators concluded no effect. |
| Joint pain | 1. Pain improvement. | 1. Effects observed in vitro testing. 2. Effects observed in animal studies. 3. Theoretical references to potential effects. 4. Simultaneous reports of positive and negative results. | 1. No research exists, and the effect is considered unlikely, even theoretically. 2. Comparative trials on direct effect indicators concluded no effect. |
| Hypertension | 1. Reduction in systolic blood pressure. 2. Reduction in diastolic blood pressure. | 1. Effects observed in vitro testing. 2. Effects observed in animal studies. 3. Theoretical references to potential effects. 4. Simultaneous reports of positive and negative results. | 1. No research exists, and the effect is considered unlikely, even theoretically. 2. Comparative trials on direct effect indicators concluded no effect. |

**Examples of how the effectiveness of dietary supplements was classified as A, B, or C.**

**1. The effectiveness of Aojiru on Hypertension was classified as C.**

The original website in Japanese.


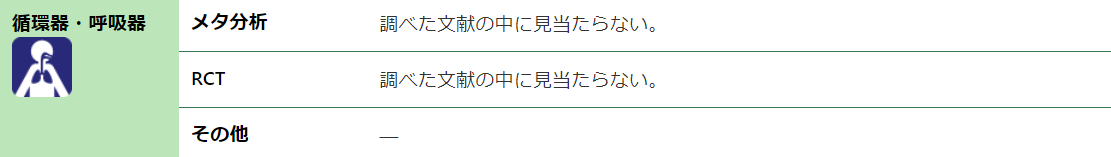


The translation in English.

| **Circulatory / Respiratory System** | **Meta-analysis** Not found in the reviewed literature. |
| --- | --- |
|  | **RCT** Not found in the reviewed literature. |
|  | **Other —** |

**2. The effectiveness of Soy Isoflavone on Diabetes was classified as B.**

The original website in Japanese.


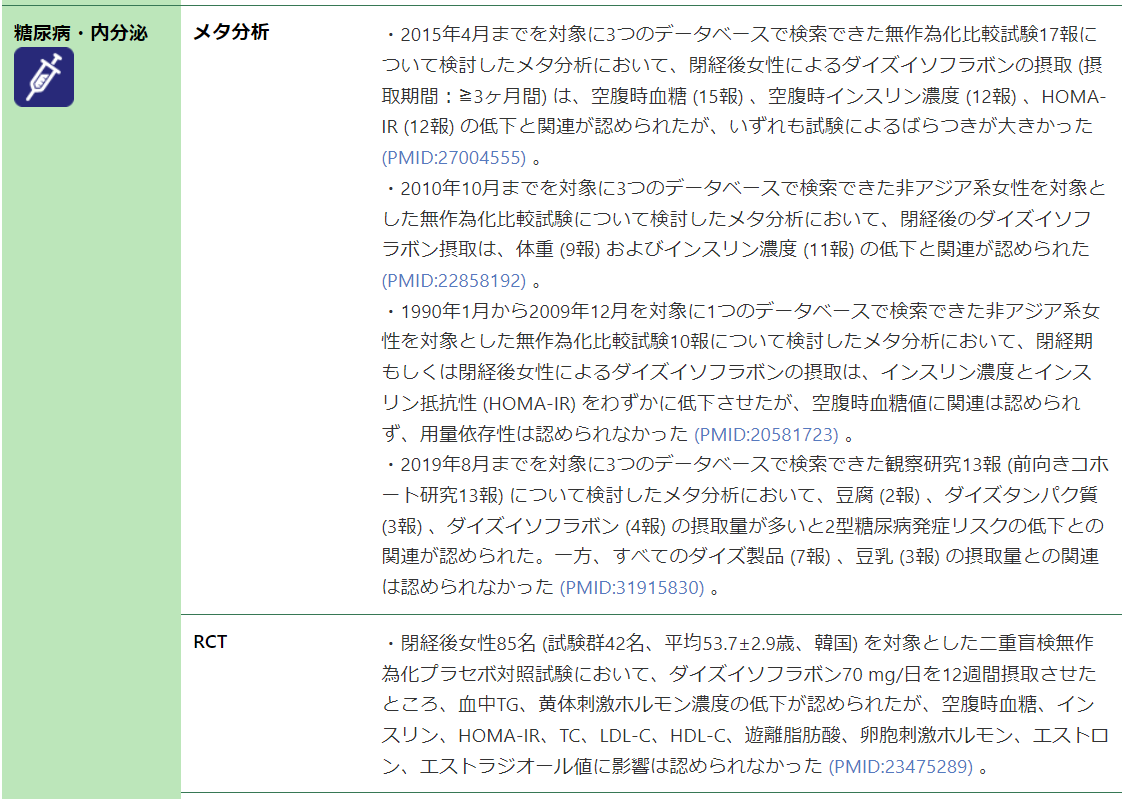


The translation in English.

| **Diabetes / Endocrine System** | **Meta-analysis**   - A meta-analysis up to April 2015 examining 17 RCTs found that intake of soy isoflavones by postmenopausal women (≥ 3 months) was associated with reductions in fasting blood glucose (15 studies), fasting insulin levels (12 studies), and HOMA-IR (12 studies), although the magnitude of effect varied widely across trials. (PMID: 27004555) - A meta-analysis up to October 2010 targeting non-Asian postmenopausal women found that soy isoflavone intake was associated with reductions in body weight (9 studies) and fasting insulin concentrations (11 studies). (PMID: 22858192) - A meta-analysis covering January 1990 – December 2009 of 10 RCTs involving non-Asian women showed slight reductions in insulin concentrations and insulin resistance (HOMA-IR) with soy isoflavone intake, but no effect on fasting glucose; dose dependency was weak. (PMID: 20571233) - A meta-analysis up to September 2019 including 13 cohort studies (2 on tofu, 3 on soy protein, 4 on soy isoflavones, 7 on total soy foods) found that higher soy or isoflavone intake was linked to lower risk of type 2 diabetes in some studies, but not in others. (PMID: 31915830) |
| --- | --- |
|  | **RCT** A randomized, double-blind, placebo-controlled trial among 85 postmenopausal women (42 in intervention, mean 53.7 ± 2.9 years, conducted in Korea) investigated 12-week supplementation of soy isoflavone 70 mg/day. Results showed decreased triglycerides and luteinizing hormone levels, but no changes in fasting glucose, insulin, HOMA-IR, total cholesterol, HDL-C, LDL-C, follicle-stimulating hormone, or estradiol. (PMID: 23475289) |

**3. The effectiveness of Turmeric on Joint pain was classified as A.**

The original website in Japanese.


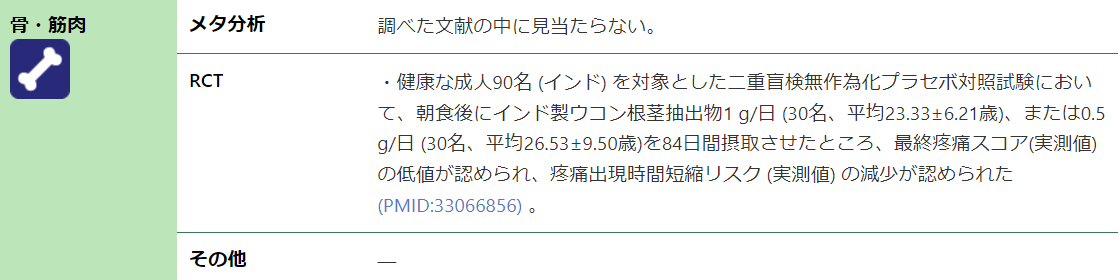


The translation in English.

| **Bone / Musculoskeletal System** | **Meta-analysis**  Not found in the reviewed literature. |
| --- | --- |
|  | **RCT** A randomized, double-blind, placebo-controlled trial conducted in India among 90 healthy adults. Participants were assigned to receive Indian-made *ukon* (turmeric) rhizome extract at either 1 g/day (30 participants, mean age 23.33 ± 6.21 years) or 0.5 g/day (30 participants, mean 26.53 ± 9.50 years) for 84 days. The trial reported a reduction in final pain scores (measured values) and a shortened risk period for the appearance of pain (measured values). (PMID: 33066856) |
|  | **Other —** |

**The guideline used to evaluate the effectiveness of dietary supplements for specific diseases as presented in the GPT responses**

| **Category** | **Definition** | **Examples** |
| --- | --- | --- |
| **A** | The text consists of statements affirming the direct or indirect effects of the supplement. No mention of denying effectiveness.  - | - Some studies show effectiveness.  - There are indirect effects.  - There are still few studies demonstrating effectiveness, but it is promising. |
| **B** | The text consists of ambiguous statements, such as both positive and negative statements about the effect, or insufficient scientific evidence for the effect, although the effect is positive. | - Mentions both effectiveness and lack of effectiveness.  - Acknowledges indirect effects but questions direct effects.  - There is some scientific evidence of effectiveness, but it is insufficient or not fully established.  - There are individual differences.  - The independent effect is limited, but when combined with treatments, it may be effective (supplementary effect). |
| **C** | The text consists of statements that question or do not mention the effect on the disease. | - It is not effective, but it is important for health.  - Effectiveness is questioned.  - Scientific evidence of effectiveness is limited.  - It does not have an effect on constipation, but it may improve gut health.  - There is little evidence that it relieves joint pain, but it helps maintain joint health. |

**An example of two physicians disagreeing on the effectiveness of DS responded by ChatGPT.**

**Original text in Japanese.**

青汁は、一般的に便秘に対して効果があるとされています。以下の理由で便秘の改善に役立つ可能性があります： 1. **食物繊維の豊富さ**: 青汁は多くの場合、ケールや大麦若葉などの食物繊維を多く含む野菜を原料としています。食物繊維は腸の運動を促進し、便のかさを増やすことで便秘を改善する助けになります。 2. **栄養バランスの向上**: 青汁にはビタミンやミネラルが豊富に含まれており、全体的な栄養バランスを改善することで腸内環境を整える効果が期待できます。 3. **水分補給**: 青汁を摂取することで、水分も同時に摂ることができます。水分不足は便秘の一因となるため、水分補給は重要です。 4. **プレバイオティクス効果**: 青汁に含まれる食物繊維は、腸内の善玉菌の餌となり、腸内フローラを整える効果が期待できます。 ただし、青汁だけでなく、バランスの取れた食事や適度な運動、十分な水分摂取など、総合的な生活習慣の改善が便秘対策には重要です。また、青汁の効果は個人差があるため、効果が感じられない場合や逆に体調に変化があった場合は、医師に相談することをお勧めします。

**Translated text in English.**

Aojiru (green vegetable juice) is generally considered effective for relieving constipation. It may help improve bowel movements for the following reasons:

1. Rich in dietary fiber:
   Aojiru is typically made from vegetables such as kale or young barley leaves, which are rich in dietary fiber. Dietary fiber promotes intestinal movement and increases stool bulk, helping to relieve constipation.
2. Improved nutritional balance:
   Aojiru contains abundant vitamins and minerals that can improve overall nutritional balance and help maintain a healthy intestinal environment.
3. Hydration:
   Drinking aojiru also provides water intake. Because dehydration is a common cause of constipation, adequate fluid intake is important.
4. Prebiotic effect:
   The dietary fiber in aojiru serves as food for beneficial gut bacteria, helping to maintain a healthy gut microbiota.

However, not only aojiru but also a well-balanced diet, moderate physical activity, and sufficient water intake are important for preventing constipation. In addition, the effect of aojiru may vary between individuals; if no improvement is felt or if adverse changes occur, consulting a physician is recommended.

**In this case, one physician judged that ChatGPT’s response indicated that aojiru was effective for relieving constipation, whereas the other physician considered it “uncertain” because ChatGPT added at the end that the effect of aojiru may vary among individuals. However, after re-discussing the case in accordance with the established guideline, both physicians agreed that ChatGPT’s answer affirmed the effectiveness of aojiru for constipation.**
