# Supplementary material for: Validity and reliability of ChatGPT's responses on dietary supplements in Japan: A quality assessment and content analysis
Source: PEC Innov. 2026 Feb 21;8:100461. doi: 10.1016/j.pecinn.2026.100461 (PMC12955162; doi:10.1016/j.pecinn.2026.100461)
Supplement: Supplementary file 3 — Supplementary material 3 [file mmc3.docx]

**Supplementary Materials 1. Evidence-based effects of 30 DS for six diseases.**

| DS name | Cancer | Diabetes | Obesity | Constipation | Joint pain | Hypertension |
| --- | --- | --- | --- | --- | --- | --- |
| Aojiru | C | C | C | C | C | C |
| Agaricus | B | B | C | C | C | C |
| Ginkgo Biloba | C | C | C | C | C | B |
| Turmeric | C | C | C | C | A | C |
| Ornithine | C | C | B | C | C | C |
| Oyster Extract | C | C | C | C | C | C |
| Chitin and Chitosan | C | C | B | C | C | B |
| Glucosamine | B | C | C | C | B | C |
| Black Vinegar | C | C | C | C | C | A |
| Chlorella | B | C | C | C | C | C |
| Korean Ginseng | B | B | C | C | C | C |
| Coenzyme Q10 | B | C | C | C | C | B |
| Collagen | C | C | C | C | B | C |
| Squalene | C | C | C | C | C | C |
| Chinese softshell turtle | C | C | C | C | C | C |
| Soy Isoflavone | B | B | B | C | C | B |
| DHA & EHA | B | B | B | C | C | B |
| Lactic acid bacteria | C | C | B | C | C | C |
| Garlic | B | B | C | C | B | B |
| Hyaluronic acid | C | C | C | C | A | C |
| Vitamin E | B | C | C | C | C | C |
| Vitamin C | B | C | C | C | C | B |
| Placenta | C | C | C | C | C | C |
| Blueberry & Bilberry | C | B | C | C | C | C |
| Prune | C | C | C | C | C | C |
| Propolis | C | B | C | C | C | C |
| Maca | C | C | C | C | C | C |
| Euglena | C | C | C | C | C | C |
| Royal jelly | C | B | C | C | C | B |
| Calcium | B | B | B | C | C | C |
