# Supplementary material for: Validity and reliability of ChatGPT's responses on dietary supplements in Japan: A quality assessment and content analysis
Source: PEC Innov. 2026 Feb 21;8:100461. doi: 10.1016/j.pecinn.2026.100461 (PMC12955162; doi:10.1016/j.pecinn.2026.100461)
Supplement: Supplementary file 4 — Supplementary material 4 [file mmc4.docx]

**Supplementary materials 4. Examples of original text in Japanese included in content analysis.**

*Content Example 1 (Translated text in English):*

*"(First Section)*

*Garlic may have some potential effects on obesity and weight management. Below are its possible effects:*

*(Second Section)*

*Boosting metabolism: Components in garlic may enhance metabolism, making it easier for the body to burn more calories.*

*Appetite suppression: The strong flavor of garlic may help reduce appetite, potentially preventing overeating and naturally lowering food intake.*

*Inhibiting fat accumulation: Some studies suggest that garlic might inhibit fat accumulation and help lower body fat percentage.*

*Supporting healthy weight management: Garlic contains many nutrients that support overall health and may be beneficial as part of a weight management plan.*

*(Third Section)*

*However, the effects of garlic on obesity and weight management heavily depend on individual lifestyle and overall diet. It's important not to rely solely on garlic but to maintain a balanced diet, regular exercise, and sufficient sleep as part of a healthy lifestyle. For diet and weight management, consulting with a nutritionist or physician is recommended. Garlic can play a supplementary role but should be considered as part of an overall diet strategy."*

*Content Example 1 (Original text in Japanese):*

*“（第一セクション）*

*ニンニクが肥満やダイエットに対してある程度の効果を持つ可能性があります。以下に、ニンニクの可能な効果を挙げます：*

*（第二セクション）*

*代謝の促進：ニンニクに含まれる成分が代謝を高める効果を持つ可能性があります。代謝が活発になると、体はより多くのカロリーを消費しやすくなります。*

*食欲抑制：ニンニクの強い風味が食欲を減少させる効果をもたらす場合があります。これにより、過食を防ぎ、食事の量を自然に減らすことができるかもしれません。*

*脂肪の蓄積抑制：一部の研究では、ニンニクが脂肪の蓄積を抑制し、体脂肪率の低下を助ける可能性が示唆されています。*

*（第三セクション）*

*健康的な体重管理のサポート：ニンニクは全体的な健康をサポートする多くの栄養素を含んでおり、健康的な体重管理プランの一部として役立つかもしれません。*

*しかし、肥満やダイエットに対するニンニクの効果は、個人の生活習慣や全体的な食事に大きく依存します。また、ニンニクだけに頼るのではなく、バランスの取れた食事、定期的な運動、十分な睡眠など、健康的なライフスタイルを維持することが重要です。ダイエットや体重管理に関しては、栄養士や医師と相談することをお勧めします。ニンニクは補助的な役割を果たすことはできますが、全体的なダイエット戦略の一部として捉えるべきです。”*

*Content Example 2 (Translated text in English):*

*"Garlic may have some potential effects on obesity and weight management."*

*Content Example 2 (Original text in Japanese):*

*“ニンニクが肥満やダイエットに対してある程度の効果を持つ可能性があります。”*

*Content Example 3 (Translated text in English):*

*" Black vinegar contains antioxidants that are beneficial for general health maintenance, but it cannot be definitively stated that it has a direct effect on cancer. However, black vinegar may reduce the risk of cancer by neutralizing free radicals in the body and reducing cellular damage."*

*Content Example 3 (Original text in Japanese):*

*“黒酢には抗酸化物質が含まれており、一般的な健康の維持に有益ですが、がんに対して直接的な効果があると断言することはできません。黒酢は体内の自由基を中和し、細胞の損傷を減らすことで、がんのリスクを低減する可能性があります。”*

*Content Example 4 (Translated text in English):*

*“Various studies have examined the effects of DHA (docosahexaenoic acid) and EPA (eicosapentaenoic acid) on diabetes, but the results are sometimes contradictory, and no clear conclusions have been reached. Below are the potential effects of DHA and EPA on diabetes based on current scientific understanding:*

*Improved insulin sensitivity: Some studies suggest that omega-3 fatty acids may improve insulin sensitivity and reduce the risk of type 2 diabetes.*

*Reduction of inflammation: DHA and EPA have anti-inflammatory properties that may help alleviate chronic inflammation in diabetic patients.*

*Blood glucose control: Some studies indicate that omega-3 fatty acids may aid in blood glucose control, though other studies have not confirmed significant effects.*

*Reduction of cardiovascular risk: Diabetic patients are at higher risk for cardiovascular diseases, and DHA and EPA may support cardiovascular health and help reduce related risks.*

*Due to inconsistent research findings, a cautious approach is necessary when considering DHA and EPA as part of diabetes treatment. The optimal treatment plan for each individual should be determined in consultation with healthcare providers and based on personal health conditions and existing diabetes management strategies. Additionally, DHA and EPA intake should ideally be part of an overall healthy diet.”*

*Content Example 4 (Original text in Japanese):*

*“DHA（ドコサヘキサエン酸）とEPA（エイコサペンタエン酸）が糖尿病に及ぼす影響については、複数の研究が行われていますが、その結果は一部で矛盾している場合もあり、明確な結論には至っていません。以下は、現在の科学的理解に基づくDHAとEPAが糖尿病に及ぼす可能性のある影響です。*

*インスリン感受性の改善: 一部の研究では、オメガ3脂肪酸がインスリン感受性を改善し、2型糖尿病のリスクを減少させる可能性が示唆されています。*

*炎症の低減: DHAとEPAには抗炎症作用があり、糖尿病患者における慢性的な炎症状態の軽減に役立つ可能性があります。*

*血糖コントロール: 一部の研究では、オメガ3脂肪酸が血糖コントロールに役立つ可能性があるとされていますが、他の研究では明確な効果が確認されていません。*

*心血管リスクの低減: 糖尿病患者は心血管疾患のリスクが高いですが、DHAとEPAは心血管系の健康をサポートし、これに関連するリスクを減少させる助けになる可能性があります。*

*これらの効果については、研究結果が一貫していないため、DHAとEPAを糖尿病治療の一環として用いる際には慎重なアプローチが必要です。個々の患者にとって最適な治療法は、医療提供者と相談し、個人の健康状態や既存の糖尿病管理計画に基づいて決定することが重要です。また、DHAとEPAの摂取は全体的な健康的な食生活の一環として行うことが望ましいです。”*

*Content Example 5 (Translated text in English, mentioning the need to follow professional guidance):*

*"Therefore, when using soy isoflavones for cancer treatment or prevention, it is essential to consult thoroughly with a doctor or specialist and receive appropriate advice."*

*Content Example 5 (Original text in Japanese, mentioning the need to follow professional guidance):*

*“したがって、大豆イソフラボンをがん治療や予防の目的で使用する場合は、医師や専門家と十分に相談し、適切なアドバイスを受けることが重要です。”*

*Content Example 6 (Translated text in English, not mentioning the need to follow professional guidance):*

*"Blueberries are believed to have antioxidant properties that may help in cancer prevention. Specifically, flavonoids such as anthocyanins in blueberries play a role in neutralizing free radicals in the body and protecting cells. This suggests they may reduce the risk of developing cancer.*

*However, there is no scientific evidence that eating blueberries can directly cure cancer. For cancer prevention or treatment, a balanced diet, regular exercise, and periodic health checkups are crucial. A healthy diet, including blueberries, may contribute to reducing cancer risk, but it cannot be claimed to treat cancer on its own."*

*Content Example 6 (Original text in Japanese, not mentioning the need to follow professional guidance):*

“*ブルーベリーには抗酸化作用があるため、がん予防に役立つ可能性があるとされています。特に、ブルーベリーに含まれるアントシアニンなどのフラボノイドは、体内のフリーラジカルを中和し、細胞を保護する役割があります。これにより、がんの発生リスクを下げる可能性が示唆されています。*

*しかし、ブルーベリーを食べることで直接がんを治す効果があるという科学的な証拠はありません。がんの予防や治療には、栄養バランスの取れた食事、適度な運動、定期的な健康診断が重要です。ブルーベリーを含めた健康的な食生活は、がんのリスクを減らす一助になるかもしれませんが、単体でがんに効くとは言えません。*”
